# A qualitative study to explore the burden of disease in activated phosphoinositide 3-kinase delta syndrome (APDS)

**DOI:** 10.1186/s13023-024-03215-9

**Published:** 2024-05-18

**Authors:** Ian Hitchcock, Hanna Skrobanski, Elina Matter, Ewen Munro, John Whalen, Joanne Tutein Nolthenius, Alex Crocker-Buque, Amanda Harrington, Delphine Vandenberghe, Sarah Acaster, Kate Williams

**Affiliations:** 1 Pharming Group N.V, Leiden, The Netherlands; 2 Acaster Lloyd Consulting Ltd, London, UK; 3 Pharming Healthcare, Inc, Warren, NJ US

## Abstract

**Background:**

Activated phosphoinositide 3-kinase delta syndrome (APDS) is an ultra-rare primary immunodeficiency, with only 256 cases reported globally. This study aimed to explore the disease burden of APDS from the perspective of individuals with APDS and their caregivers.

**Methods:**

Qualitative interviews were conducted with healthcare providers (HCPs), individuals with APDS and caregivers, to explore the symptoms and health-related quality of life (HRQoL) impact of APDS. Some individuals and caregivers also completed a narrative account exercise. All interviews were audio recorded and transcribed. Data were analysed using thematic analysis and saturation was recorded.

**Results:**

Semi-structured qualitative interviews were conducted with healthcare providers (HCPs), individuals with APDS and caregivers. Individuals and caregivers had the option of completing a narrative account exercise. Six HCPs participated in an interview. Seven participants completed the narrative account exercise (*N* = 5 caregivers and *N* = 2 individuals with APDS) and 12 took part in an interview (*N* = 4 caregivers and *N* = 8 individuals with APDS). Themes identified from HCPs interviews included symptoms, clinical manifestations, HRQoL impacts and treatments/management of APDS. The narrative account exercise identified similar themes, but with the addition to the journey to diagnosis. These themes were explored during the individual/caregiver interviews. Reported clinical manifestations and symptoms of APDS included susceptibility to infections, lymphoproliferation, gastrointestinal (GI) disorders, fatigue, bodily pain, and breathing difficulties. HRQoL impacts of living with APDS included negative impacts to daily activities, including work, education and social and leisure activities, physical functioning, as well as emotional well-being, such as concern for the future, and interpersonal relationships. Impacts to caregiver HRQoL included negative impacts to physical health, work, emotional well-being, interpersonal relationships and family life and holidays. The management of APDS included the use of healthcare services and medications including immunoglobulin replacement therapy (IRT), rapamycin, prophylactic antibiotics, leniolisib, as well as medical procedures due to complications.

**Conclusions:**

APDS has a high disease burden and there is an unmet need for licensed, more targeted treatments which modify disease progression. This study was the first to describe the day-to-day experience and HRQoL impact of APDS from the perspective of individuals living with the condition, caregivers and treating physicians.

**Supplementary Information:**

The online version contains supplementary material available at 10.1186/s13023-024-03215-9.

## Background

Activated phosphoinositide 3-kinase delta syndrome (APDS) is an ultra-rare primary immunodeficiency (PID) first characterised in 2013, with only 256 cases reported globally [1–5]. The disease is categorised into two types depending on the causal mutation, with APDS1 associated with the PIK3CD mutation, and APDS2 with the PIK3R1 mutation [2, 6]. Conventional medical interventions for APDS include preventative antibiotics, immunoglobin replacement therapy (IRT), immunosuppressive therapies and haematopoietic stem cell transplant (HSCT) [1, 3, 4, 6]. However, most of these interventions are not disease modifying agents and are primarily focused on managing and treating the symptoms of APDS. HSCT is potentially curative but is accompanied by the risk of complications (such as Graft-versus-Host Disease) and associated mortality [7]. Additionally, despite the historically available treatments, individuals with APDS experience the risk of a reduced lifespan with the most common causes of death being lymphoma and complications resulting from HSCT [8]. Most recently, leniolisib (CDZ173), an oral inhibitor of the p110δ subunit of PI3Kδ, was approved by the United States (US) Food and Drug Administration (FDA) in March 2023, and is the first approved medication for the treatment of APDS [6, 9].

Commonly cited manifestations of APDS include, but are not limited to, recurrent respiratory tract infections [2, 3, 6, 10–12], chronic lung diseases such as bronchiectasis and asthma [13, 14], herpes virus infections, gastrointestinal (GI) disorders, and autoimmune and autoinflammatory disorders, as well as chronic non-malignant lymphoproliferation including generalised lymphadenopathy and hepatosplenomegaly [2–4, 6, 10]. Additionally, individuals with APDS are at greater risk of developing haematologic malignancies such as diffuse Large B cell lymphoma [4] and may have growth and neurodevelopmental abnormalities [3, 10, 15]. However, to date, no studies have explored the HRQoL impacts of these symptoms and manifestations on individuals or caregivers.

Qualitative research enables an in-depth exploration of a disease's impact, allowing participants to share their experiences beyond the constraints of closed-question surveys or questionnaires. This is particularly valuable in rare diseases, such as APDS, as it can highlight previously unknown symptoms and impacts. No qualitative studies have previously been conducted with individuals with APDS or their caregivers. Therefore, the primary aim of this study was to qualitatively explore the symptoms, impacts and challenges experienced by individuals with APDS, as well as experience with treatment for APDS. The secondary aim was to explore the experience of caring for and treating an individual with APDS.

## Methods

### Design and participants

The main component of this study included qualitative interviews of individuals with APDS and caregivers. These were informed by healthcare provider (HCP) interviews and a narrative account exercise, completed by a selection of individuals with APDS/caregivers prior to their interview. To take part in the study, individuals had to have a confirmed diagnosis with APDS and be aged 12 years or older. Caregivers were eligible to participate if they were a parent or other caregiver providing ≥ 50% care to an individual with a confirmed diagnosis of APDS and were willing and able to consent to take part. The only exclusion criterion for caregivers was paid caregivers. HCPs were eligible if they were currently a registered nursing professional or licensed physician, with experience working with at least one individual with APDS.

### Study materials

Three semi-structured interview guides were developed for the individual/caregiver interviews, which were tailored to either 1) adults with APDS, 2) adolescents with APDS or 3) caregivers. The guides were developed based on findings from a targeted literature search, the HCP interviews, and the individual/caregiver narrative accounts. The guides included open-ended questions on symptoms, treatments and impacts of living with APDS or caring for someone with APDS, including perceptions of meaningful change in symptoms and impacts.

An HCP interview guide, background questionnaires for HCPs, individuals and caregivers, and instructions for the narrative account exercise with individuals and caregivers, were also developed.

### Ethical approval

The study was reviewed and approved by Western Institutional Review Board (WIRB-Copernicus Group Independent Review Board (IRB tracking number: 20226879).

### Recruitment and data collection

Recruitment took place through a specialist recruitment agency between January and June 2023. HCPs were sought from Canada, France, Germany, Italy, Spain, and the United Kingdom (UK). Individuals APDS and caregivers were sought from Australia, Canada, France, Germany, Italy, Spain, the Czech Republic, the Netherlands, the Republic of Ireland, the UK, and the United States (US). HCPs were invited to participate in the study by the recruitment agency via email. Individuals with APDS and caregivers were either invited by email (if they were part of a patient database) or direct social media message, or responded to a recruitment advertisement. Potential patients were asked to contact the recruitment agency using the contact details provided. Adult individuals/caregivers and parents/legal guardians of adolescent individuals gave written informed consent to participate or their child to participate in the study, while adolescent individuals gave written assent. Most caregiver participants included in the study were not attached to study participants who had APDS. However, three caregivers took part alongside their child in the interviews, and another caregiver took part in the narrative account while their child subsequently took part in the interview alone. It was not known whether HCPs were attached to an individual with APDS in the study as this information was not collected, and HCPs and individuals with APDS were recruited independently.

All participants received an information sheet about the study alongside a background questionnaire to complete and return by email. A selection of individuals and caregivers were invited to participate in the narrative account exercise prior to an interview. This involved asking participants to write or record a voice note with an unstructured detailed account of their experience of APDS and return via email.

HCPs interviews were conducted in February 2023, and individual/caregiver interviews were conducted between February and June 2023. Individuals and caregivers re-confirmed their consent/assent verbally at the start of each interview. All interviews were conducted over teleconference, lasted approximately 60 min, and were audio-recorded. All HCP interviews, and the English language individual/caregiver interviews were conducted in English by two study authors (HS and EM), both with an MSc or PhD in Psychology and more than 13 years combined qualitative research experience. The non-English individual/caregiver interviews were conducted by trained interviewers in each study country in the local language. Some parents/legal guardians of adolescent individuals were present during interviews when requested by the care recipient. None of the participants were known to the interviewers.

Recordings were transcribed verbatim, and non-English transcripts and written accounts were translated into English. All transcripts and written accounts were de-identified and assigned participant identification numbers.

### Analysis

Quantitative data collected from the background questionnaires were summarised using descriptive statistics in Excel [16]. Qualitative data collected from the narrative accounts and interviews with individuals with APDS, caregivers and HCPs were analysed separately using thematic analysis in MAXQDA [17].

Two researchers (HS and EM) independently developed and tested an initial coding framework on the same section of a transcript or narrative account. For each dataset, the coding was compared and an inter-coder agreement on the final frameworks were reached before the entire narrative accounts or transcripts were coded. A senior researcher, (KW) also reviewed the initial and revised coding frameworks to enhance the quality of the analysis. Concepts were identified from the coded segments from each dataset and grouped into themes. Data saturation for the individual /caregiver interviews was monitored using a saturation grid, with concepts in columns and interview participants as rows.

A conceptual model was developed using the concepts identified from the individual/caregiver interviews to provide a visual representation of the burden of living with APDS and the relationships between concepts.

## Results

### Sample characteristics

Six HCPs with experience treating individuals with APDS participated in an interview. All were licensed physicians, with clinical specialties in immunology (*n* = 3; [50.0%]), haematology (*n* = 2; [33.3%]) and internal medicine (*n* = 1; [16.6%]). Physicians’ years of experience in the role ranged from 19–32 years, and years of experience specifically treating individuals with APDS ranged from 3–15 years. Four HCPs had experience with treating five or more individuals with APDS.

Seven participants completed the narrative account exercise (*N* = 5 caregivers and *N* = 2 individuals with APDS), at which point enough information was collected to inform the development of the interview guides. Twelve participants took part in an interview (*N* = 4 caregivers and *N* = 8 individuals with APDS). Tables 1 and 2 provide more detail on the demographic and clinical characteristics of individuals with APDS.

**Table 1:** Demographic characteristics of individuals with APDS

Characteristic	*N* = 12^*a*^
Age, years	
Mean (SD)	19.8 (15.2)
Min, max	0.8, 56.0
Gender, *n* (%)	
Female	9 (75.0)
Male	3 (25.0)
Country, *n* (%)	
US	8 (66.7)
UK	2 (16.7)
Spain	1 (8.3)
Australia	1 (8.3)
Employment status, *n* (%)	
In education or training	6 (50.0)
Employed full-time	1 (8.3)
Unable to work due to health	3 (25.0)
Other	2 (16.7)
Highest level of education, *n* (%)	
Still in school	6 (50.0)
Secondary education, high school or equivalent	3 (25.0)
University/college degree or equivalent	2 (16.7)
Other	1 (8.3)

^a^Sum of counts includes both individuals with APDS who participated in the study themselves (*N* = 8) as well as those whose caregivers participated on their behalf (*N* = 4)

**Table 2:** Clinical and treatment characteristics of individuals with APDS

Characteristic	Total (*N* = 12)^a^
**Age first noticed signs of APDS, years (*N* = 9)**^**b**^	
Mean (SD)	4.2 (3.5)
Min, max	0.0, 9.5
**Age first sought medical help for symptoms/issues related to APDS, years (*N* = 11)**	
Mean (SD)	2.0 (2.0)
Min, max	0.0, 7.0
**Age at diagnosis, years (*N* = 11)**	
Mean (SD)	11.6 (9.6)
Min, max	0.8, 35.0
**APDS type, *n* (%)**	
APDS 1	5 (41.7)
APDS 2	5 (41.7)
Don’t know/unsure	2 (16.7)
**Health conditions or complications ever experienced, *n* (%)**	
Infections	12 (100)
Lymphoproliferation	9 (75.0)
Digestive tract issues	7 (58.3)
Autoimmune or autoinflammatory disease	3 (25.0)
Lymphoma	2 (16.7)
Developmental disorders	9 (75.0)
**Treatment with leniolisib, *n* (%)**	
Currently treated	2 (16.7)
Never treated	10 (88.3)

^a^Sum of counts includes both individuals with APDS who participated in the study themselves (*N* = 8) as well as those whose caregivers participated on their behalf (*N* = 4)

^b^Sum of counts is less than 12 as three participants provided either a vague response (*n* = 2) or no response (*n* = 1)

### Healthcare professional (HCP) interview findings

HCPs reported a range of symptoms and clinical manifestations associated with APDS, including susceptibility to infections (*n* = 6), lymphoproliferation (*n* = 6), GI issues (*n* = 5), autoimmune and autoinflammatory disorders (*n* = 5), neuropsychiatric disorders (*n* = 5) and fatigue (*n* = 2). Variations in symptoms and clinical manifestations based on clinical status (e.g. more active lymphoproliferation in more advanced individuals with APDS) (*n* = 4), and disease progression (e.g. malignancies) (*n* = 3), were also discussed. HCPs also noted variations with age (*n* = 3), with a higher prevalence of recurrent respiratory infections in paediatric individuals with APDS. In contrast, adults with APDS were reported to have more frequent or severe clinical manifestations (e.g. more gastrointestinal issues) (*n* = 3). HCPs reported perceived impacts on individuals with APDS, including work and school (*n* = 3), daily and social activities (*n* = 5) and emotional well-being (*n* = 3). Three HCPs perceived the HRQoL of their individuals with APDS as low (*n* = 2) or moderate (*n* = 1).

All six HCPs discussed immunoglobulin replacement therapy (IRT) as a treatment for APDS, including intravenous immunoglobulin (IVIG) and subcutaneous immunoglobulin (SCIG) therapies. Reported benefits of IRT included its affordability and accessibility (*n* = 1), and its perceived impact on improving quality of life and reducing bacterial infections. One HCP reported that they avoid prescribing it due to the risk of adverse drug reactions which could be fatal. Reported side effects of IRT included headaches (*N* = 1), body aches (*N* = 1), chills (*N* = 1), fever (*N* = 1), malaise (*N* = 1), local pain at the injection site (*N* = 1) and fatigue (*N* = 1).

Four HCPs reported on their experience treating patients with leniolisib. Potential benefits of leniolisib included its perceived impact on reducing infection frequency (*N* = 1), diminishing lymphoproliferation (*n* = 3), improving fatigue (*N* = 1) and improving clinical biomarkers (*N* = 3), such as immunoglobulin levels. However, one HCP also expressed concerns about the lack of long-term safety data for leniolisib, and another reported that one of their patients had experienced a temporary elevation in hepatic enzymes, aspartate transaminase (AST) and alanine transaminase (ALT).

Perceived attributes of a successful treatment in general included stopping disease progression (*n* = 2), improvements in clinical biomarkers (*n* = 2), a reduction in infection frequency and associated symptoms (*n* = 2), and improvements in lymphoproliferation (*n* = 1). All HCPs (*n* = 6) discussed the need for targeted treatments for APDS. One HCP discussed that a key attribute of a successful treatment is for the patient to regain their quality of life.

*“…the main goal for anyone I’m treating with APDS is to return, to retain and [be] able to gain back their quality of life that they’ve been missing on because of the impact of this illness on their life.” - HCP 201, Haematologist, UK*

Additional example quotes describing the key insights from the HCP interviews are provided in Additional Material 1.

### Individual with APDS and caregiver narrative account exercise findings

All participants (*N* = 7) provided a written account, ranging between 1–4 pages in length. The main themes raised were: (1) Journey to diagnosis; (2) Clinical manifestations and symptoms of APDS; (3) Impacts on APDS patient’s HRQoL; (4) Caregiver HRQoL impacts and family HRQoL impacts; and (5) APDS management. Example quotes from the narrative account exercise describing the perspective of individuals with APDS and their caregivers are provided in Additional Material 2.

### Individual with APDS and caregiver interview findings

**Fig. 1:**
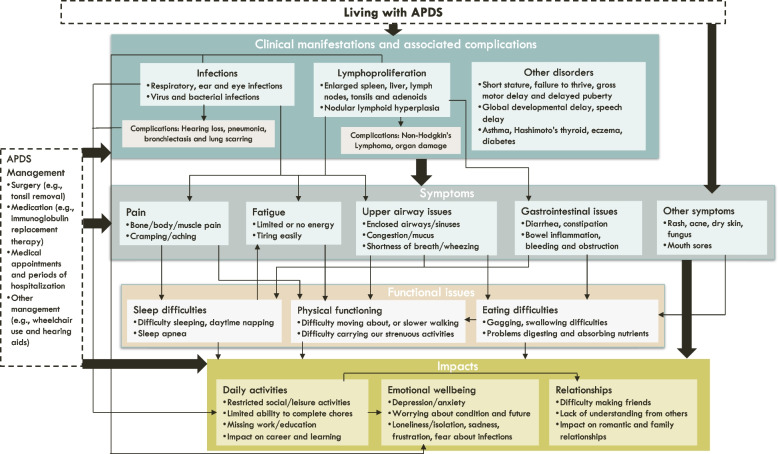
Conceptual model of the experience of APDS. The top line of the conceptual model underneath the ‘living with APDS’ box, shows the clinical manifestations of APDS and associated complications. The second line shows the symptoms associated with APDS which were described to be caused by different clinical manifestations or to be general symptoms of the condition and the third row shows functional issues that were described to be caused by the symptoms of APDS rather than to be a direct consequence of APDS or associated clinical manifestations. The final row shows the HRQoL impacts reported to be associated with clinical manifestations, symptoms, and functional issues as well as management of APDS. Arrows show reported relationships between concepts

The main themes identified from the interviews included clinical manifestations, symptoms, functional issues, HRQoL impacts and APDS management. The relationships between these themes are shown in a conceptual model (Fig. 1). Data saturation was reached for the broad overarching themes, with 78.1% of the individual with APDS concepts and 57.1% of the caregiver concepts reported in the first five interviews (Table 3).

**Table 3:**
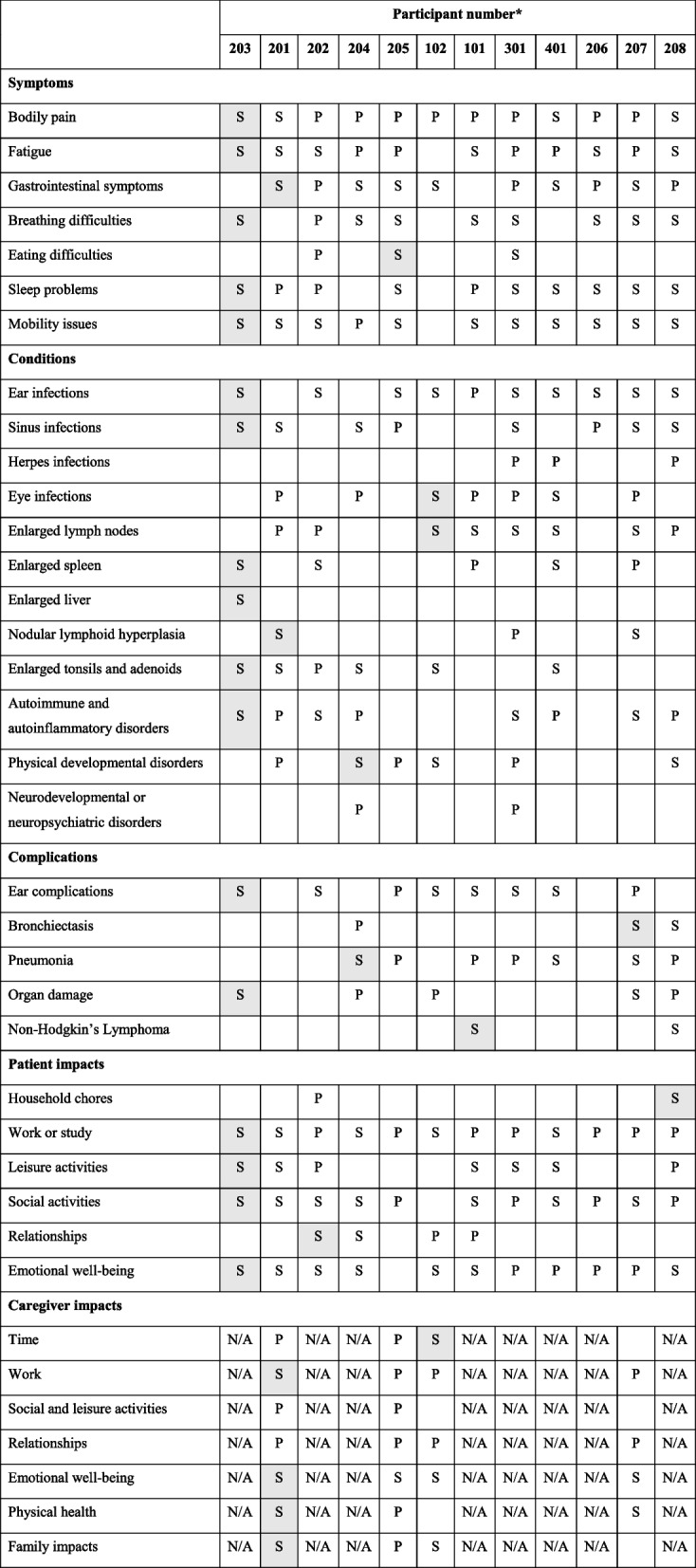
Saturation matrix

*N/A* Caregiver concept not applicable as participant was an individual with APDS, *S* Spontaneously reported (shaded cell is first spontaneous report), *P* Probed

^a^Participant numbers are listed in the order interviews were conducted

### Clinical manifestations and associated symptoms of APDS

All participants (*n* = 12) reported susceptibility to infections as a clinical manifestation of APDS. Types of infections included ear infections (*n* = 10), sinus infections (*n* = 8), eye infections (*n* = 7), fungal infection (*n* = 1), and respiratory infections and associated complications such as pneumonia (*n* = 7), colds (*n* = 3), flu (*n* = 3), bronchitis (*n* = 2) and bronchiectasis (*n* = 3). Participants described various forms of lymphoproliferation, including enlarged nymph nodes (*n* = 8), spleen (*n* = 5), tonsils (*n* = 4), adenoids (*n* = 3) and liver (*n* = 1) as well as nodular lymphoid hyperplasia (*n* = 3). Other disorders associated with APDS reported by participants were autoimmune or autoinflammatory disorders (*n* = 8), physical developmental disorders (*n* = 6), blood coagulation disorders (*n* = 3), anaemia (*n* = 2), kidney and liver disease (*n* = 1) and thrombocytopenia (*n* = 1). Participants reported the various impacts of these additional disorders, including difficulty in movement (*n* = 2) due to their legs being different lengths (*n* = 1) or low muscle mass (*n* = 1) and concerns around future family planning because of delayed puberty (*n* = 2).

APDS and its clinical manifestations were reported to be associated with a range of symptoms, including bodily pain (*n* = 12), fatigue (*n* = 11), GI symptoms (*n* = 10) such as diarrhoea (*n* = 7) and constipation (*n* = 6), and breathing difficulties (*n* = 9). Other symptoms reported by more than one participant included skin rashes (*n* = 4) and mouth sores (*n* = 4).

### Impact of clinical manifestations and symptoms on daily life and HRQoL

Participants reported that bodily pain (*n* = 7), fatigue (*n* = 6) and breathing difficulties (*n* = 3) all impaired their physical functioning, including their ability to stand, walk and climb stairs without assistance:

*“There was a point where I had to be in a wheelchair and on oxygen because my lungs weren’t able to work by themselves.” - Participant 203, 16-year-old individual with APDS, US*

Further, participants reported that fatigue (*n* = 8), breathing difficulties (*n* = 5), GI disorders (*n* = 3) impacted their daily activities, including their ability to work, and partake in housework and social and leisure activities, and their sleep. For example, one individual with APDS stated that their GI disorders would require them always to be near a toilet:

*“I felt like I couldn’t even go do anything because I had to always be around the toilet, like I couldn’t get in the car because I was too scared I would have to go to the bathroom.”- Participant 204, 28-year-old individual with APDS, US*

Some participants reported lymphoproliferation impacted their sleep (*n* = 3) and their ability to eat (*n* = 1) due to associated breathing and swallowing difficulties.

*“She would end up choking and gagging on stuff because she couldn’t swallow very well because of the tonsils” - Participant 201, caregiver of 3-year-old individual with APDS, US*

Several participants described how infections, or the risk of infections, impacted their / their care recipients’ lives such as restrictions to their social and leisure activities due to the need to shield. Six participants reported regular absences from work or school due to illness resulting in gaps in learning or career development.

*“I had gaps in learning because I was off ill. I think science and maths said to mum one parents’ evening that, ‘She’ll be here one lesson, she’ll then be off one or two lessons and then she’ll be back but she misses that link between the two lessons, so she’ll get one lesson and get the other one but she’ll miss the middle lesson link’ so I didn’t do very well in certain subjects because I didn’t have the links” - Participant 101, 24-year-old patient, UK.*

Seven participants reported emotional impacts, such as the fear of socialising, and loneliness due to having to shield themselves from potential infections.

*“I fear going to prom, I fear going to graduation, I fear going to a lot of big events because I worry about what I might catch and if I’ll come back from it” - Participant 202, 17-year-old individual with APDS, US*

Other emotional impacts included concerns for the future (*n* = 6) including worry about disease progression (*n* = 3), loneliness (*n* = 4), anxiety (*n* = 3), depression (*n* = 3), frustration (*n* = 2) and feeling downhearted (*n* = 1).

*“I worry about lymphoma, especially since I’m predisposed to the lymphoma cancer, and for me it can at times become very overwhelming” - Participant 208, 56-year-old patient, US*

Likewise, four participants reported impacts to their interpersonal relationships because of APDS, which mostly derived from a lack of understanding of APDS by their peers (*n* = 3).

*“When everybody found out that we had an immunodeficiency, APDS, they thought that it was contagious. We were barred from ever being friends with anybody” - Participant 202, 17-year-old individual with APDS, US.*

### Perceived impact of improved symptoms on HRQoL

Participants described how an improvement in symptoms, such as, GI disorders (*n* = 5 out of 6 asked), fatigue (*n* = 11 out of 12 asked), bodily pain (*n* = 7 out of 7 asked) and breathing difficulties (*n* = 1 out of 1 asked) would have a meaningful impact on their lives.

*“It would be nice for her to enjoy a family vacation. We’re taking her to Disney, and that was where we went last time, and she didn’t really get to enjoy it much. She slept through half of the princesses that she wanted to see.” – Participant 201, caregiver of 3-year-old individual with APDS, US*

Six of nine participants asked, reported that an improvement in susceptibility to infections would have a meaningful impact on individuals’ lives, attributed to improvements in their ability to carry out daily activities such as school, work, and social and leisure activities, as well as recovery from infections and frequency of medical appointments.

*“It would be more meaningful if I could spend more time with my friends without having to worry about getting as sick” - Participant 204, 28-year-old individual with APDS, US*

Two individuals (*n* = 10 not asked) stated an improvement to lymphoproliferation would be meaningful to their HRQoL due to reduced pain and worry about developing cancer.

*“It would be very meaningful, not having to worry about the lumps, not having to worry about those lumps turning into lymphoma, the pain, obviously.” - Participant 401, 38-year-old individual with APDS, Australia*

### Management of APDS

Participants described their prolonged journey to diagnosis (*n* = 7), taking three to 22 years duration from when they first developed symptoms to receiving an official diagnosis of ADPS. Two caregivers expressed the negative emotional impact of having to wait for their care recipient’s illness to be diagnosed. Participants also reported negative experiences of using healthcare services for APDS, such as HCPs dismissing their concerns (*N* = 1) and HCP’s lack of understanding about APDS restricting their ability treat the condition (*N* = 3).

*“I think sometimes [HCPs] get to a point where they don’t know what else to do to help [daughter] and so then we get pushed down on the totem pole as far as people to call back or respond to or follow up with because they kind of hit a road block and don’t know how else to help” - Participant 201, caregiver of 3-year-old individual with APDS, US*

Participants discussed the frequency of medical appointments as every six months (*n* = 4), once a month (*n* = 2) or a couple times a week (*n* = 1). Likewise, the reported time taken for medical appointments ranged from two to three hours (*n* = 4) to a couple of days (*n* = 1). Further, most participants (*n* = 10) reported that they (or their care recipient) had experienced emergency hospital admission relating to APDS, for reasons such as having pneumonia (*n* = 1) and experiencing fever (*n* = 1) and shingles (*n* = 1). Participants also mentioned the use of prophylactic antibiotics to prevent infections (*n* = 7) and other types of antibiotics in response to developing infections (*n* = 5).

For participants with experiences with IRT (*n* = 11), ten reported that IRT had a positive effect on reducing APDS symptoms. However, eight of these participants also reported side effects, including headaches (*n* = 3), pain at infusion site (*n* = 3), fatigue (*n* = 2), itch (*n* = 1), nausea (*n* = 1), kidney pain (*n* = 1) and kidney issues (*n* = 1).

Participants reported various impacts of APDS management, including frequent or prolonged time off work or school (*n* = 8), the need to plan medical care before leaving home (*n* = 5) and concern for the future due to the impact of frequent IRT administration (*n* = 1).

*“The intravenous immunoglobulin [intravenous IRT] has brought my look on life a little to the breaking point because I struggle with, “Well who’s going to cover this if I get a job? What am I going to do when I do have to go off to college? Where am I going to see my nurse?” - Participant 202, 17-year-old individual with APDS, US*

Two participants had experience with leniolisib (*n* = 2). One individual with APDS, who had received leniolisib for over four years, reported improvements such as their tonsils not growing back again after a tonsillectomy, stabilising lung capacity and breathing, fewer periods of hospitalisation and illness. They also reported gaining weight but viewed this as a disadvantage. The other participant, a caregiver, reported no changes in their 12-year-old adolescent care recipient’s symptoms or impacts since they started leniolisib four months previously; however, they questioned their care recipient’s dosage strength. The caregiver also reported that their care recipient (not a participant) experienced bouts of extreme tiredness in the initial stages of treatment.

Participants reported the characteristics of a successful treatment, including improved symptoms (*n* = 6) and clinical outcomes (*n* = 2), living a normal life (*n* = 2) as well as no need (*n* = 1) or a reduced dosage (*n* = 1) for medication.

### Caregiver HRQoL impacts

Four caregivers described the HRQoL impacts of caring for someone with APDS. Three reported spending a substantial time providing care, which included managing referrals and medical appointments (*n* = 4), administering medication (*n* = 3), complicated medical procedures (*n* = 2), ordering medical supplies (*n* = 2), research on APDS (*n* = 1) and managing insurance (*n* = 1).

*“It’s really hard sometimes. Learning how to do quite a complicated medical procedure that has to be done in a sterile way. There’s specialist skills that you have to develop in terms of needles and all that kind of thing” - Participant 102, caregiver of 11-year-old individual with APDS, UK*

Three caregivers reported impacts to their physical health, including physical tension (*n* = 1), fatigue (*n* = 1), and sleep difficulties (*n* = 1) due to the stress of caring for a child with APDS.

*“I was just in such worry for the child that I’d sometimes get up in the night just to make sure she was okay or worrying when I’d go to work. My physical health I’d say was just more sleep deprived” - Participant 205, caregiver of 10-month-old individual with APDS, US*

All caregivers (*n* = 4) reported impacted daily activities which mostly pertained to work, including reduced working hours (*n* = 2) or quitting their job completely (*n* = 1) to cope with caring responsibilities. One caregiver reported missing out on employment advancement opportunities and conflict with their co-workers.

*“I’ve possibly missed some employment advancement opportunities because of my absence and it also can cause a bit of strife with co-workers who happen to pick up my end of the workload while, in my absence.” - Participant 205, caregiver of 10-month-old individual with APDS, US*

Moreover, all caregivers reported an impact to their emotional well-being, including reports of loneliness and isolation (*n* = 2), anxiety (*n* = 2), depression (*n* = 1), low moods (*n* = 1) and feelings of stress (*n* = 1), irritability (*n* = 1) and hopelessness (*n* = 1).

Finally, three caregivers reported an impact to their family life, which included restrictions to family holidays due to risk of infection (*n* = 1) or logistical concerns around treatment schedule (*n* = 1) or medical equipment (*n* = 1). Two caregivers reported avoiding leisure activities to shield due to the patient’s risk of infections.

*“We don’t get together with people like we used to, just because either A, it’s not exposing her to other stuff, or we’re too tired to or she just is not feeling up to it, and so then we cut that out.” - Participant 201, caregiver of a 3-year-old individual with APDS, US*

## Discussion

This was the first qualitative study exploring the impact of APDS from the perspective of individuals with APDS and caregivers. It provides several insights into the humanistic burden of the disease. While clinical studies have previously reported symptoms, functions, and clinical manifestation of APDS, this study augments this by highlighting the specific impacts that these have on the daily life and HRQoL of individuals living with the condition [9, 18, 19]. Living with APDS was reported to substantially impact the HRQoL of individuals, including work and education, social and leisure activities, household chores, physical functioning, relationships, and emotional well-being, such as concern for the future. Participants also highlighted the burden experienced by individuals with APDS associated with attending frequent medical appointments, having numerous periods of hospitalisation, and organising and planning around their medical care (e.g., IRT administration). Participants additionally described how the prolonged journey to APDS diagnosis, and their perceptions that HCPs lack of knowledge about this ultra-rare condition, added to their emotional burden.

The conceptual model expands these findings by illustrating the relationship between the clinical manifestations, symptoms, functional issues, and impacts. The interconnectivity of these factors highlights how an improvement or worsening in one area has a knock-on effect on other symptoms and areas of life. For instance, based on the model, an improvement in lymphoproliferation has the potential to lead to improvements in symptoms and functional issues, such as pain, breathing difficulties, and physical function, which may in turn lead to improvements in HRQoL impacts, such as worry about developing lymphoma and ability to take part in leisure and social activities.

The study also highlighted caregiver burden in APDS. Identified caregiver impacts included impacts on daily activities (particularly involving work), emotional well-being, interpersonal relationships, and physical health. Family impacts were also reported, such as restricted ability to partake in social and leisure activities as a family or going on family holidays due to life evolving around the individual with APDS’s needs.

Although this was the first qualitative study exploring the HRQoL of individuals with APDS, its findings are consistent with studies investigating the HRQoL of individuals with other PIDs. Compared to healthy individuals, people with PIDs experience significantly lower HRQoL [20]. Studies have shown that HRQoL in children with primary antibody deficiencies similar to APDS are often worse than the HRQoL of children with other chronic conditions [21, 22].

Some limitations were present within this study. Firstly, although participants were sought from across Australia, Canada, the US and Europe, the majority were recruited from the US, potentially impacting the transferability of the results to other individuals with APDS and caregiver populations. However, there is no published evidence to suggest that individuals with APDS and their caregivers within these countries would experience HRQoL vastly differently from those within the US and further research is needed to confirm the transferability of these results across study populations. Additionally, some volunteer and non-response bias may be present within the study sample, as recruitment was determined by participants’ volition. Individuals who volunteer for a study may possess distinct characteristics compared to those that do not. However, qualitative research is not designed to be representative, and, as APDS is an ultra-rare disease, recruitment relied on a limited pool of individuals agreeing to participate. The data saturation matrix for the individual/caregiver interviews indicated that saturation had been reached, with most concepts first being mentioned spontaneously within the first five interviews. However, given the heterogeneity in the reported clinical manifestations, symptoms and functional issues, additional interviews may have identified new concepts. It is also worth noting that the insights gathered from the caregivers interviewed on behalf of individuals with APDS relied purely on observation and may not have directly reflected their care recipient's experience.

## Conclusion

This is the first study to describe the day-to-day experience and HRQoL impact of this ultra-rare condition, APDS, from the perspective of individuals, and their caregivers, as well as the perspective of treating physicians. The findings highlight a high disease burden and the substantial unmet need in this population for more licensed and targeted effective treatments which modify disease progression, and that are less burdensome than conventional treatments (e.g., oral medication). Moreover, the findings emphasise the need for better awareness and understanding of the symptoms and manifestations of APDS, including any differences between paediatric and adult patients, to limit the risk of misdiagnosis and shorten the time to referral to an immunologist to obtain a diagnosis and initiate effective treatment options.

### Supplementary Information

Additional file 1: Additional Material 1. HCP Interview findings. Exemplary quotes from HCPS describing their experiences with APDS.

Additional file 2: Additional Material 2. Patient/Caregiver narrative account findings. Exemplary quotes from patients and caregivers describing their experiences with APDS.

## Data Availability

Raw data (interview transcripts) are not publicly available to protect participant privacy, as APDS is a rare disease and there are few participants in each country.
