# Supplementary material for: A qualitative study to explore the burden of disease in activated phosphoinositide 3-kinase delta syndrome (APDS)
Source: Orphanet J Rare Dis. 2024 May 18;19:203. doi: 10.1186/s13023-024-03215-9 (PMC11102230; doi:10.1186/s13023-024-03215-9)
Supplement: Supplementary file 1 — Additional file 1: Additional Material 1. HCP Interview findings. Exemplary quotes from HCPS describing their experiences with APDS. [file 13023_2024_3215_MOESM1_ESM.docx]

**Additional Material 1 – HCP interview findings**

**Exemplary quotes from HCPs describing their experiences with APDS.**

| **Topic** | **Quote** |
| --- | --- |
| **Clinical manifestations/symptoms** |  |
| Respiratory infections | *“Lung infections and upper respiratory tract infections, that, that’s the majority of people [with APDS]” – HCP 301, haematologist, UK* |
| Viral infections | *“The most APDS related viruses are EBV, CMV and also HPV” - HCP 401, Haematologist, France* |
| Bacterial infections | *“Bacterial or virus, ocular infection, conjunctivitis, digestive, uh, campylobacter genus, salmonella, clostridium" - HCP 201, Haematologist, Spain* |
| Eye infections | *“Infection like for example … chronic conjunctivitis" - HCP 101, Immunologist, Italy* |
| Lymphoproliferation | *“That impacts their appearance, I’ve seen some patients who had these lymph nodes it even affects their clothing… [they try] to hide it so their appearance can, for them it’s not pleasant but that’s only if it’s something that can be visible…it can impact them psychologically” - HCP 301, Haematologist, UK*  *“The tonsils at the back of our throat they are a certain size, when they become very large, they can of course, can affect…the ability to breather properly, sleeping at night is impacted, sleep apnoea, um, it becomes a source of greater infection because it’s larger, it’s trapping more food particles” – HCP 501, immunologist, US* |
| Gastrointestinal (GI) issues | *“Digestive disease because there is going to be a lot of enteropathy and malabsorption, and this also leads to failure to thrive because there is not enough nutrients or energy.” – HCP 401, internal medicine, France.*  *“Enteropathy is diarrhoea, fever, colic pain that, that is very invalidating for the patient and also problems of uh, of absorption of the nutrients.” – HCP 201, haematologist, Spain* |
| Autoimmune and autoinflammatory disorders | *“All those things where the immune system is turning against itself and attacking its own organs, become very, very serious and a very major complications of this disorder.” – HCP 501, Immunologist, US* |
| Fatigue | *“The fatigue…is very impacting…they are always lacking of energy” – HCP 401, Haematologist, France* |
| Neurodevelopmental and  neuropsychiatric disorders | *“At 15 years-old or 20 years-old, then comes the real psychiatric problems like anxiousness and depression” (HCP 401, Haematologist, France)* |
| **Variation in symptoms and clinical manifestations** |  |
| Differences between APDS 1 and APDS 2 | *“Both have infections, respiratory infection but perhaps APDS2 have more cytopenia, and not so good response to rapamycin” – HCP 201, Haematologist, Spain* |
| Variation in clinical manifestations by age | *“I think early on just very classic with the recurring ear infections, pharyngeal infections and then respiratory tract infections, and often accompanied by cough, very wet cough, um with a lot of wheezing and dyspnoea.” – HCP 501, Immunologist, US* |
| Distinctions between more and less advanced  APDS | *“The severe ones, that is – a patient who actually needs to be hospitalised for these infections so something that has not been treated in the primary setting with an oral treatment” – HCP 301, Haematologist, UK* |
| Signs of progression from less advanced APDS  to more advanced APDS | *“The number of organs involved so you’re getting more than one, for example, beyond just the respiratory tract, now they’re getting involvement of GI tract, neurological impact and then, the need for greater and greater therapy.” – HCP 501, US, Immunologist* |
| **Perceived HRQoL impacts** |  |
| Daily and social activities | *“If somebody’s got a cough and she wants to go to sleepovers, she’s not going to the sleepover and that’s not the experience of most 14 year old girls” – HCP 601, Immunologist, Canada* |
| Emotional wellbeing | *“Psychologically as well, especially if, they know that it’s something that maybe could have been picked up on earlier on and treated maybe more effectively.” – HCP 301, Haematologist, UK.* |
| Work and Study | *“These type of patients…cannot study very well, cannot frequent a regular period in a school---the complexity of the, the disease is linked to its comorbidities and the quality of life of these patients is very low.” – HCP 101, Immunologist, Italy* |
| Biggest impact on QoL | *“Young people…with no possibility of a normal life---in job or in studying or in social---to go to the cinema or to go---to dance.” – HCP 201, Haematologist, Spain* |
| Family life | *“Normally, they are a young person, so father, mother, sister or a couple, need to take care of the patient in some of cases and come to the hospital and stay in the hospital, hospitalisation, so the quality of life of the person that takes care of these patients are also impaired … because they lose jobs” - HCP 201, Haematologist, Spain* |
| **Treatments** |  |
| Immunoglobulin Replacement Therapy (IRT) | *“The treatment with the immunoglobulin therapy like rituximab because it has shown one of the most, the best efficiency in disease control with the less infection and overall survival.” – HCP 401, internal medicine, France*  *“The most common ones they get, there’s headache, body ache, chills and even some fever. There can be some malaise that can occur for a day or two after, possibly, they feel a bit out of sorts.” HCP 501, Immunologist, US* |
| Leniolisib | *“The tolerability of the product has been very promising, we know the science behind it they are able to measure markers as we’ve discussed earlier, to see that it’s actually doing something and they’re seeing diminution in the lymph node and, and spleen size so all the stuff we talked about is actually addressed by this, so it’s very, very exciting.” – HCP 501, Immunologist, US*  *“They [clinical trial subjects] improve in response, in you know, improve in [the] control [of] the disease, improve in reducing the lymphadenopathy, in reduce the percent, the percentage of patients that develop a lymphoma so uh, it’s a …very well received for the patients, well tolerate[d].” – HCP 201, Haematologist, Spain* |
| Prophylactic antibiotics | *“Even the patients [that don’t] have a lot of infections during the year and not a lot of symptoms, we’ll still put them with antibiotic prophylaxis and see if it can, this can manage to control the disease” - HCP 401, Haematologist, France* |
| Immunosuppressive drugs | *“Immunosuppressive drug in the cases of massive lymphoproliferation, for [example] sirolimus” - HCP 101, Immunologist, Italy* |
| Hematopoietic stem cell transplantation (HSCT) | *“I think the bone marrow transplant I think that can be considered maybe for a younger patients like in their teens or maybe even younger than that, like children” - HCP 501, Immunologist, US)* |
| Treatment choice by disease severity | *“If there is not a lot of, less severity or less, low risk then antibiotics prophylaxis is just enough but the higher the risk or the higher the symptoms, then you have to be aggressive with the treatment” - HCP 401, Haematologist, France* |
| Patient’s tolerability of treatment | *“So ‘tolerate treatment’ and efficacy of treatment are two different things. She could be on death’s doorstep and she’d tolerate the treatment that she’s on, right? If we’re talking about the efficacy of the treatment then yeah, how severe you are, you might need, you might need more” - HCP 601, Immunologist, Canada* |
| **Perceptions of a successful treatment** | *“I think the most important is to reduce the number of recurrent and opportunistic infections during one year for these patients because the comorbidities and the mortality for these patients is linked to the complexity of the lung disease, or the gastrointestinal infectious disease” – HCP 101, Immunologist, Italy*  *“There’s adequate levels of antibodies that prevent patients from having recurrent infections, unable to carry on with their life, erm, with, without erm, without the impact of the disease so these are the, the main goal, the main goal for anyone I’m treating with APDS is to return, to retain and able to gain back their quality of life that they’ve been missing on because of the, the impact of this illness on their life.”- HCP 201, Haematologist, UK* |
| **Unmet need for targeted treatments** | *“All these other [treatments] I just talked about are sort of band aids or covering the problem but not really treating the problem specifically. So I really believe the selective inhibitors in my mind are probably the most important and certainly at the most cutting edge so if we give something that can actually impact this overactive gene, then you’re treating the problem at the root and that’s where I think the future lies with this condition – and other conditions too” – HCP 501, Immunologist, US.*  *“I believe that the most efficient treatments in every disease are the most specific and the most disease related treatment, not general treatment” – HCP 401, Haematologist, France*  *“The future perhaps is this: targeted therapy for moderate, severe and perhaps also in mild, to try to avoid the problems that we can have later, it’s a very small group of patients, the price could be high but the government have a special budget for these rare diseases so in my opinion, these new treatments like leniolisib…need to be the first line of treatment to try to avoid complications” – HCP 201, Haematologist, Spain* |
