# Supplementary material for: A qualitative study to explore the burden of disease in activated phosphoinositide 3-kinase delta syndrome (APDS)
Source: Orphanet J Rare Dis. 2024 May 18;19:203. doi: 10.1186/s13023-024-03215-9 (PMC11102230; doi:10.1186/s13023-024-03215-9)
Supplement: Supplementary file 2 — Additional file 2: Additional Material 2. Patient/Caregiver narrative account findings. Exemplary quotes from patients and caregivers describing their experiences with APDS. [file 13023_2024_3215_MOESM2_ESM.docx]

**Additional Material 2 – Patient/Caregiver narrative account findings**

**Example quotes from patients and caregivers describing their experiences with APDS.**

| **Topic** | **Quote** |
| --- | --- |
| **Journey to diagnosis** |  |
| First signs/symptoms | *“From about the age of 2 my daughter had symptoms which we now understand to be linked to APDS but was yet to be diagnosed. These included persistent coughs, colds and mucus from the nose leading to her being scheduled for a tonsillectomy and adenoidectomy plus grommets insertion at 2.5 years old.” - P102, caregiver of 11-year-old patient, UK* |
| Difficulties in receiving a diagnosis | *“They just brushed my illness and infections off as normal childhood disease. My mother had to be my advocate to get anything done for me medically. My parents ended up making a VHS recording of my coughing fits and that got the doctors’ attention enough to send me to a local hospital - P204, 28-year-old patient, US* |
| Time taken to receive a diagnosis | *“Bear in mind, she was born in 2005 so we could continue to struggle with figuring out what exactly is causing her to be so sick for another 8 to 9 years” – P202, caregiver of 17-year-old patient, US* |
| Impact of HCP’s limited information | *“I eventually learned to live with it but I was essentially just herded into a group of unknown immunodeficiency due to medical professionals not being able to understand what was wrong with me due to the lack of information about APDS and the human genome” - P204, 28-year-old patient, US* |
| **Clinical manifestations and symptoms** |  |
| Respiratory infections | *“Some symptoms my child experiences are frequent infections such as sinus and ear infections along with recurrent pneumonia” – P205, caregiver of 10-month-old patient, US* |
| Lymphoproliferation | *“My 17-year-old has had a history of enlarged liver and spleen, chronic upper respiratory infections, especially her ears.” - P202, caregiver of 17-year-old patient, US* |
| General symptoms | *“I also feel the constant lack of energy, tiredness, muscle aches and cold-like symptom, through something I can’t change, becomes more of an annoyance than a frustration after a while.” – P101, 24-year-old patient, UK* |
| **HRQoL Impacts** |  |
| Emotional wellbeing | *“She is having such a hard time emotionally trying to figure out what’s next for her life living with the unpredictability of APDS and what issue is going to pop up next.” – P202, caregiver of 17-year-old patient, US*  *“Emotionally she struggles; she knows she has an extremely rare condition; she’s never met anyone with the same condition and she feels isolated.” – P102, caregiver of 11-year-old patient, UK* |
| Daily activities | *“It didn’t take long for me to realise I couldn’t hold down a typical full-time job. At around 18 years old I decided to go self-employed in the desperate attempt of reaching my childcare dream. Again, this didn’t last very long due to being around young children I was often sick. After being signed off for over 6 months I was deemed medically unable to work” – P101, 24-year-old patient, UK*  *“APDS also affects my social life as an adult. I have to constantly worry about getting sick and I try to limit the amount of people I come in contact with in order to prevent infection as best as possible” – P204, 28-year-old patient, US* |
| Relationships | *“Due to the lack of understanding in society I often struggled to fit in with my peers. I felt judged by peers just because teachers and other adults gave me a little leeway in regard to homework and stuff.” – P101, 24-year-old patient, UK* |
| **Caregiver HRQoL Impacts** |  |
| Emotional wellbeing | *“Emotionally it can be very tough; knowing that her condition comes from a genetic mutation; knowing there is no cure; seeing her struggle with the impact on her life and choices; knowing she will have to deal with this her whole life and it impacts on her choices in the future (50% chance of passing to the next generation); feeling anxious about her contracting infections; felling anxious about how her condition may affect her in the future; knowing some of the risks” – P102, caregiver of 11-year-old patient, UK* |
| Physical impacts | *“I feel more tired during the day as well due to constantly caring for my child with APDS” - P205, caregiver of 10-month-old patient, US* |
| Daily activities | *“It makes life more difficult to do any social activities, go to work, engage in any hobbies, or plan any events. I always need to be able to be with my child during illnesses and for appointments” – P205, caregiver of 10-month-old patient, US* |
| Financial impacts | *“Because of the increased doctor appointments, time needed to advocate for her, coordination of appointments, medications, and supplies, my income has dropped by approximately 50%. This has caused an additional strain on our family but also her sibling who has become limited in what we can do as a family” - P201, caregiver of 3-year-old patient, US* |
| **Family HRQoL Impacts** |  |
| Burden of planning social arrangements around  healthcare needs | *“Life with APDS has caused additional challenges when travelling with a child who requires us to bring a lot of additional medications and feeding supplies. In addition, we really limit the amount of germs that she is exposed to since she has a harder time fighting them off” P201, caregiver of 3-year-old patient, US* |
| Impact of care recipient’s siblings | *“As far as her brother he’s 8. He knows mostly that we deal with more than a “normal” family does. He does have anxiety and separation issues. He doesn’t like to be alone. It’s always hard on him when we have to travel to his sister at the hospital” – P203, caregiver of 16-year-old patients, US* |
| **APDS Management** |  |
| Treatments | *“Since all my symptoms started at such a young age, I was not able to have a normal childhood life. I constantly attended doctor appointments and had tests, IVs, labs and more that I thought were a cruel punishment” - P204, 28-year-old patient, US* |
| Additional management and medical  procedures | *“I had a tonsillectomy and adenoidectomy at age 5 and had my tonsils removed again around age 11 due to them growing back from APDS.” - P204, 28-year-old patient, US* |
| Healthcare use | *“Over the years she’s had 40 procedures/surgeries. Countless hospitalisations, ear infections, upper respiratory infections, staph, pneumonia, and Covid” – P203, caregiver of 16-year-old patient, US* |
